# Annotations

**Published:** 1904-09-03

**Authors:** 


### Annotations.

### What is a Post-Graduate?

New circumstances sometimes need new terms to describe them, and the process of invention is not always wisely guided. Of recent years much has been heard of the provision of facilities for further professional study for those who have completed their student curriculum and passed into the medical profession through one or other of the appointed portals. In some quarters it has seemed necessary to invent a new term to describe those who avail themselves of such facilities. Formerly, the term "graduate," or "diplomate," or "medical practitioner," was considered sufficient, but for some reason or other the authorities in charge of certain courses of instruction for practitioners have invented and continue to use the term "post-graduate." Have then the persons to whom this term is applied ceased to be graduates and passed into some later stage of blessedness on the one hand, or misery on the other? Between these alternatives we shall not attempt to discriminate, and even the announcement in one of the college prospectuses that "a reading-room and smoking-room are reserved for the use of post-graduates" does not alter our decision. What, however, is certain is that if words mean anything, "post-graduate" as a descriptive term is an absurdity. The only persons (if any) to whom it could be legitimately applied would be those, if such there be, who, having once held a University degree, have had it taken from them or have voluntarily renounced it. But, applied to medical practitioners engaged in systematic study at a hospital or college, it is ridiculously inappropriate. That a period of study or some other form of experience may follow graduation and may thus be described as "post-graduation" in point of time is, of course, beyond dispute. But "post-graduate" is both horrible in form and inaccurate in substance. It is to be hoped that certain college authorities, having managed to provide the "smoking-room," will now find time and opportunity to attend to the elements of etymology.

### Underfed School Children.

The statement, to which Sir John Gorst has recently lent the weight of his authority, that a not inconsiderable percentage of children attending the public elementary schools in England are systematically underfed, is obviously a very serious one. To compel a child to attend school and to perform its allotted tasks is a duty which the local educational authorities cannot avoid, but there is no provision to secure that the physical condition of the children is maintained at an adequate level. Only in very gross cases indeed can parents be brought under the discipline of the criminal law. Obviously, the state of matters now endorsed by a former Minister of Education must be remedied in some way or other. To drive children through the strain of scholastic exercises whilst they are receiving insufficient food is nothing short of systematic cruelty, and will not be tolerated by the public conscience. But the position is one which raises large and very difficult questions. To replace the parent by the State as the guardian of the children is far from the policy which commends itself to the public opinion of this country. Such a suggestion, or even the germ of it, would be decisively negatived by popular vote, and in this the parental voice itself would be most emphatic. Again, to supply gratis meals to improperly nourished children would be to put a premium upon careless, ignorant, and wicked parents, and would be a proceeding open to the most serious abuse. Moreover, the ratepayers, if this were undertaken at the public charge, would have something to say on the matter. The most hopeful proposal is that the teacher—though he would surely need adequate medical and other support—shall have power to give dinner tickets to underfed children, the charges being recovered by public authority from the parents. The mere risk of the exposure entailed by such a proceeding would exercise a wholesome terror over the unnatural parent, as cruelty to children carries social censure in all classes of the population. But, however remedied, it cannot be tolerated that children can be by public action compelled to perform tasks for which they are physically unfit.

### The "Index Medicus."

It would not be easy to name a periodical of greater value to the present generation of medical men than the *Index Medicus.* With but slight difficulty and little expenditure of time it is possible to follow in its pages the progress in every branch of medicine and surgery as shown by the literature appearing from month to month in the civilised countries of the world. In spite of its great merits, the preparation of this work is costly, while from its nature it is hardly likely to obtain a great circulation, being more in demand as a library unit than as a journal to which a large proportion of individual medical practitioners will subscribe. Chiefly for these reasons the *Index Medicus* has had a chequered existence. On more than one occasion it has apparently breathed its last, only to be resuscitated for another short period of activity. On the last occasion it was the Carnegie Institution in Washington who came to the rescue, and who in the early part of 1903 revived the work after it had been suspended for nearly four years. It will be remembered that the Carnegie Institution promised an annual gift of £2,000 for three years for carrying on the work, and intimated that at the expiration of this probationary period the grant would be discontinued unless there was sufficient evidence of the utility of the work as shown by the number of copies subscribed for. Unfortunately some "strikes" in the printing offices at Boston caused a delay in the issue of the number which deals with the literature of January of this year, and this delay resulted in the distribution of a circular letter emanating from the *Institut de Bibliographie Médicale de Paris* to the effect that the publication of the *Index Medicus* would probably not be resumed this year. We are very glad to know that this rumour is unfounded, and that at the worst we may look forward to receiving the *Index Medicus* up to the end of 1906, as promised by the Carnegie Institution. As the 12 annual parts of the work can be obtained for the small sum of 25s. a year, it is greatly to be hoped that the medical profession will not be slow to show their appreciation of the Carnegie Institution's offer, and by subscribing in sufficient numbers for the *Index Medicus* to ensure its issue for many years to come.

